# Characterization of Whole Blood Gene Expression Profiles as a Sequel to Globin mRNA Reduction in Patients with Sickle Cell Disease

**DOI:** 10.1371/journal.pone.0006484

**Published:** 2009-08-03

**Authors:** Nalini Raghavachari, Xiuli Xu, Peter J. Munson, Mark T. Gladwin

**Affiliations:** 1 Pulmonary and Vascular Medicine Branch, National Heart Lung and Blood Institute, NIH, Bethesda, Maryland, United States of America; 2 National Heart Lung and Blood Institute (NHLBI) Genomics Core Facility, National Heart Lung and Blood Institute, NIH, Bethesda, Maryland, United States of America; 3 Mathematical and Statistical Computing Lab, Division of Computational Biosciences, Center for Information Technology, NIH, Maryland, United States of America; 4 Pulmonary, Allergy and Critical Care Medicine and Hemostasis and Vascular Biology Research Institute in University of Pittsburgh Medical Center, Pittsburgh, Pennsylvania, United States of America; Baylor College of Medicine, United States of America

## Abstract

Global transcriptome analysis of whole blood RNA using microarrays has been proven to be challenging due to the high abundance of globin transcripts that constitute 70% of whole blood mRNA. This is a particular problem in patients with sickle cell disease, secondary to the high abundance of globin-expressing nucleated red blood cells and reticulocytes in the circulation. In order to accurately measure the steady state blood transcriptome in sickle cell patients we evaluated the efficacy of reducing globin transcripts in PAXgene stabilized RNA for genome-wide transcriptome analyses using microarrays. We demonstrate here by both microarrays and Q-PCR that the globin mRNA depletion method resulted in 55–65 fold reduction in globin transcripts in whole blood collected from healthy volunteers and sickle cell disease patients. This led to an improvement in microarray data quality by reducing data variability, with increased detection rate of expressed genes and improved overlap with the expression signatures of isolated peripheral blood mononuclear (PBMC) preparations. Analysis of differences between the whole blood transcriptome and PBMC transcriptome revealed important erythrocyte genes that participate in sickle cell pathogenesis and compensation. The combination of globin mRNA reduction after whole-blood RNA stabilization represents a robust clinical research methodology for the discovery of biomarkers for hematologic diseases.

## Introduction

Discovery of transcriptional biomarkers represents a promising strategy in the field of translational medicine for early disease detection, the development of personalized therapy for complex diseases, and for the definition of disease specific signaling pathways [1], [2]. In this regard, microarray based transcriptome analysis appears to be a frontier technology for the identification of potential biomarkers by application to biological materials most relevant to the phenotypes under investigation. These include biopsy materials from fine needle aspirates (FNA), cell sub-populations, or enriched isolates from laser capture microdissection (LCM). Although transcriptional profiles in such target disease tissues/cells are ideal for such analyses, the complexity of procuring such tissue biopsies/cells and the low amount of RNA from these specimens for standard microarray assays has made whole blood, a practical and an attractive surrogate tissue in clinical research [3], [4], [5], [6], [7]. The ready availability of blood, non/minimally invasive method of specimen collection and the critical role of blood cells in immune response out-measure the advantages of blood to tissue biopsies/cells in measuring disease state and drug response. Using a global transcriptional analysis of PBMC cells from patients with sickle cell disease we identified important disease modifying pathways, including upregulation of catalytic antioxidant (Peroxiredoxin, Glutathione peroxidase, glutathione S transferase, Thioredoxin) and anti-proliferative cell cycle regulators (Heme oxygenase 1, Biliverdin Reductase and P21 [1].

Traditionally whole blood samples for gene expression were collected in CPT tubes (Becton Dickinson) with an anticoagulant. However, RNA expression profiles have been observed to change over time in such fractionated samples [4], [8] thereby implying the need for stabilization of RNA between sample collection and isolation to maintain the expression profile of blood cells. Though this was achieved by the isolation of peripheral blood mononuclear cells immediately after specimen collection, the labor intensive techniques involved in this process and the need for technical experts in the field at the time of sample collection hinder its applicability in large scale multi center clinical trials. Attempts to overcome these hurdles led to the development of new approaches that would ease the sample collection and RNA stabilization [9]. In this respect, the PAXgene (PAX) blood RNA system has been widely employed in gene expression studies of peripheral blood. Although, this system employs an easy way to collect, store, transport and stabilize RNA from whole blood, many studies have demonstrated that RNA prepared from the PAXgene blood tubes result in significant increase in overall variability and decrease in the transcript detection sensitivity using microarrays [6]. The observed anomalies have largely been attributed to the presence of predominant amounts of globin transcripts that constitute ∼70% of mRNA in whole blood samples [7]. We found that this represents a major problem for the study of hemolytic anemias, such as sickle cell disease, owing to the high abundance of globin transcripts in nucleated erythrocytes and reticulocytes. Addressing these limitations, we undertook the current study of evaluating methods that combine stabilization of RNA and reduction of globin transcripts in whole blood to determine the suitability of the globin reduced RNA for microarray based transcriptome studies in sickle cell disease. We demonstrate herein that efficient removal of globin transcripts in PAXgene stabilized whole blood significantly improves the detection sensitivity of transcripts on microarrays and enhances the identification of genes that are significantly modulated during the sickle cell disease process. We also report here our observations on the similarities and dissimilarities of the expressed transcripts in the PBMCs and globin enriched and globin depleted whole blood. From a biological perspective, the globin depleted PAX gene versus the PBMC difference expression profile provides a window into real time erythrocyte expression profiles in sickle cell disease.

## Methods

### Patients and Control Subjects

The study was approved by the National Heart Lung and Blood Institute's institutional review board and written informed consents were obtained from study participants. Patients selected for this study included 10 patients with sickle cell disease of mean age 41.6±10.1 and the control self-identified 10 African American subjects of mean age 42.2±8.9. The patient's samples were collected in steady state condition and none were on anti-platelet medication. Expression profiles (Control N = 5; SCD N = 5) were compared to differential gene expression data generated in a prior study of 27 patients and 13 controls [1].

### Peripheral blood mononuclear cell isolation and RNA isolation

Peripheral blood from sickle cell patients, and healthy African-American volunteers, was collected into Vacutainer cell preparation tube (CPT) with sodium citrate and Ficoll (Becton Dickinson, Franklin Lakes, NJ). Purified peripheral blood mononuclear cell (PBMC) suspensions were resuspended in buffer RLT (700–1000 µL per 10^7^ cells) and passed through Qiashredder columns (Qiagen, Valencia, CA) then stored at –70° C. Total RNA was extracted from peripheral blood mononuclear cells using RNeasy Mini Kit (Qiagen).

### Isolation of RNA from whole blood specimens

Blood Specimen (2.5 ml) collected in PAXgene™ tubes from each subject was incubated at room temperature for 4 h for RNA stabilization and then stored at − 80 °C. RNA was extracted from whole blood using the PAXgene™ Blood RNA System Kit following the manufacturer's guidelines. Briefly, samples were removed from −80°C and incubated at room temperature for 2 hours to ensure complete lysis. Following lysis, the tubes were centrifuged for 10 min at 5,000×g the supernatant was discarded and 500 µL of RNase-free water added to the pellet. The tube was vortexed thoroughly to re-suspend the pellet, centrifuged for 10 min at 5000×g and the entire supernatant was discarded. The pellet was re-suspended in 360 µL of buffer BR1 by vortexing and further purification of RNA was done following the manufacturer's protocol with on-column DNase digestion.

Quality of the purified RNA from was verified on an Agilent® 2100 Bioanalyzer (Agilent Technologies, Palo Alto, CA); RNA concentrations were determined using a NanoDrop® ND-1000 spectrophotometer (NanoDrop Technologies, Wilmington, DE).

### Depletion of Globin Transcripts

Globin mRNA was depleted from a portion of each total RNA sample isolated from PAXgene tubes using the GLOBINclear™-Human kit (Ambion, Austin, TX). In brief 2 µg of total RNA from human whole blood was mixed with a biotinylated Capture Oligo Mix in hybridization buffer. The mixture was incubated for 15 minutes to allow the biotinylated oligonucleotides to hybridize with the globin mRNA species. Streptavidin magnetic Beads were then added, to capture the globin mRNA and the magnetic beads were then pulled to the side of the tube with a magnet and the RNA, depleted of the globin mRNA, was transferred to a fresh tube. The RNA was further purified using a rapid magnetic bead-based purification method as suggested by the manufacturer.

### Preparation of biotinylated cRNA targets for gene chip hybridizations

The cRNA labeling and hybridizations were performed according to protocols from Affymetrix Inc. (Santa Clara, CA). Briefly, 1 µg of total RNA from PBMC, whole blood (PAX) and Globin depleted (PAX-GR) samples was converted to double-stranded cDNA with a T7-(dT) 24 primer. The cDNA was in vitro transcribed to biotinylated complementary RNA (cRNA) by incorporating biotin-CTP and biotin-UTP using affymetrix IVT labeling kit. Twenty µg of biotin-labeled RNA was fragmented to ∼200 bp size by incubating in fragmentation buffer containing 200 mM Tris-acetate pH 8.2, 500 mM potassium acetate and 500 mM magnesium acetate for 35 minutes at 94°C prior to hybridization. Fragmented RNA was assessed for relative length on Agilent 2100 bioanalyzer and hybridized to Affymetrix HG-U133 plus 2.0 chips for 16 hours, washed, stained on an Affymetrix fluidics station and scanned using Affymetrix genechip scanner.

### Microarray Data Processing and Analysis

Affymetrix GCOS version 1.4 was used to calculate the signal intensity and the percent present calls on the hybridized Affymetrix chip. The signal intensity values obtained for probe sets in the microarrays were transformed using an adaptive variance-stabilizing, quantile-normalizing transformation termed “S10” (described in Munson, P.J., GeneLogic Workshop of Low Level Analysis of Affymetrix GeneChip Data, 2001, http://stat-www.berkeley.edu/users/terry/zarray/Affy/GL_Workshop/genelogic2001.html, software available at http://abs.cit.nih.gov/geneexpression.html.). This S10 transformation serves to make the variances uniform up and down the measurement scale, while at the same time allowing direct comparisons of transformed intensities between chips, and renders the data in a scale nearly matching the base 10 logarithm in the central range of measurement. Transformed data from all the chips were subjected to a principal component analysis (PCA) to allow for detection of outliers. Student t-test between sickle and control was performed for PBMC, PAX and PAX globin-reduced respectively and the p-value for differences between two groups was calculated for each of the 54,675 Probe Sets. To address the multiple comparisons problem, false discovery rate (FDR) was calculated for each probe set. FDR filters were applied in addition to fold change (FC) cutoff. Two-way hierarchical clustering was used to bring together sets of samples and genes with similar expression pattern. The hierarchical cluster is run from the JMP statistical software package (www.jmp.com, Cary, NC) using the ward method.

To compare this data with previously published data which was generated on HG-U95 array, array comparison data for best match from HG-U95 to HG-U133 plus 2.0 arrays downloaded from Affymetrix website were used to map probe sets between the two arrays. Gene ontology analysis was done using DAVID Bioinformatics Resources 2007 online (http://david.abcc.ncifcrf.gov/)

All the microarray data reported in this manuscript is described in accordance with the MIAME guidelines. The data has also been submitted to GEO and the submission # is GSE 16728.

### Real Time Q-PCR analysis

First-strand cDNA was synthesized using 500 ng of RNA and random primers in a 20 µl reverse transcriptase reaction mixture using Invitrogen's Superscript cDNA synthesis kit (Invitrogen, Carlsbad, CA) following the manufacturer's directions. Quantitative real-time PCR assays were carried out with the use of gene-specific double fluorescently labeled probes in a 7900 Sequence Detector (PE Applied Biosystems, Norwalk, CT). Probes and primers were obtained from Applied Biosystems. In brief, PCR amplification was performed in a 384 well plate with a 20-µl reaction mixture containing 300 nm of each primer, 200 nm probe, 200 nm dNTP in 1x real time PCR buffer and passive reference (ROX) fluorochrome. The thermal cycling conditions were 2 min at 50° C and 10 min at 95° C, followed by 40 cycles of 15 sec denaturation at 95° C and 1 min annealing and extension at 60° C. Samples were analyzed in duplicate and the C_T_ values obtained were normalized to the housekeeping gene β actin. The comparative C_T_ (ΔΔC_T_) method which compares the differences in C_T_ values between groups to achieve the relative fold change in gene expression.

## Results

### Quality of total RNA and biotinylated cRNA from PBMC, PAX and PAX-GR

**Figure 1:**
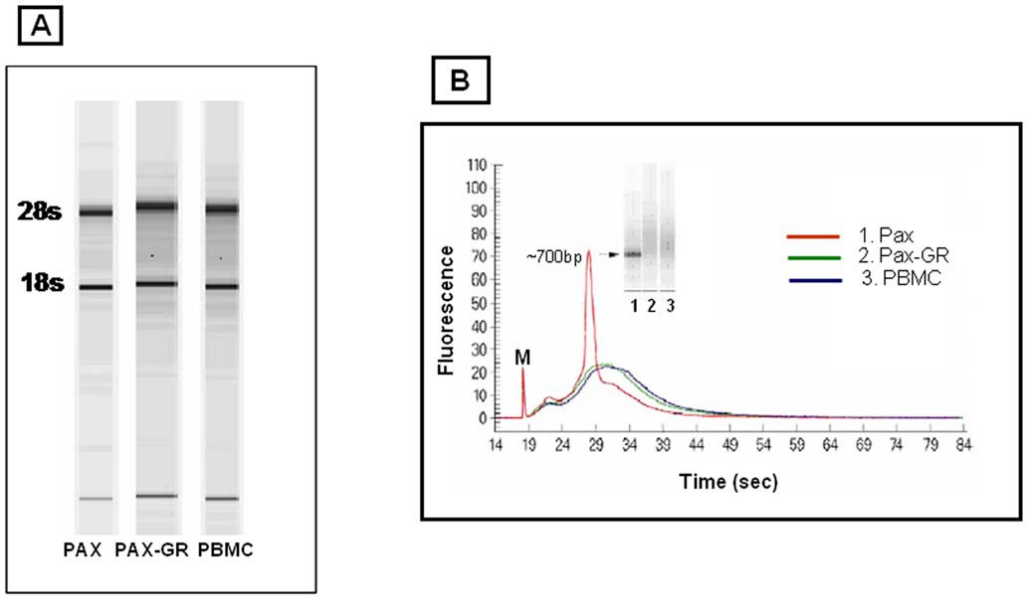
A. Quality of total RNA from PBMC, PAX and PAX-GR. The intact 18 and 28s ribosomal bands are shown in the electophoregram determined in agilent bioanalyzer. B. Quality of biotinylated cRNA from PBMC, PAX and PAX-GR. A predominant 700 bp peak represents the globin cRNA in the PAX samples. cRNA from PBMC and PAX-GR show a similar pattern of a smear of transcripts. 1. Red line - PAX. 2. Blue line - PAX- GR. 3. Green line - PBMC.

Examination of the isolated total RNA from PBMC, PAX, PAX-GR on a lab on a chip system found them to be of high quality with intact 18s and 28s ribosomal RNA with RIN numbers greater than 9.0 as shown in Figure 1A. There was about 20–25% loss of RNA during globin depletion process. 1 µg of total RNA from PBMC, PAX AND PAX-GR was processed for the synthesis of biotinylated cRNA for gene chip hybridizations. The cRNA yield from these samples were in the range of 25±4.1 with an acceptable 260/280 ratio of 1.9–2.1. On analysis using the bioanalyzer, a pronounced peak corresponding to ∼700 nucleotides representing globin cRNA was found to be present only in PAX RNA samples. The PBMC and PAX-GR samples showed identical smear pattern of cRNA products as shown in Figure 1B suggesting the depletion of substantial amounts of globins in the PAX-GR samples.

### Expression of Globin transcripts by RT-PCR and microarrays

**Figure 2:**
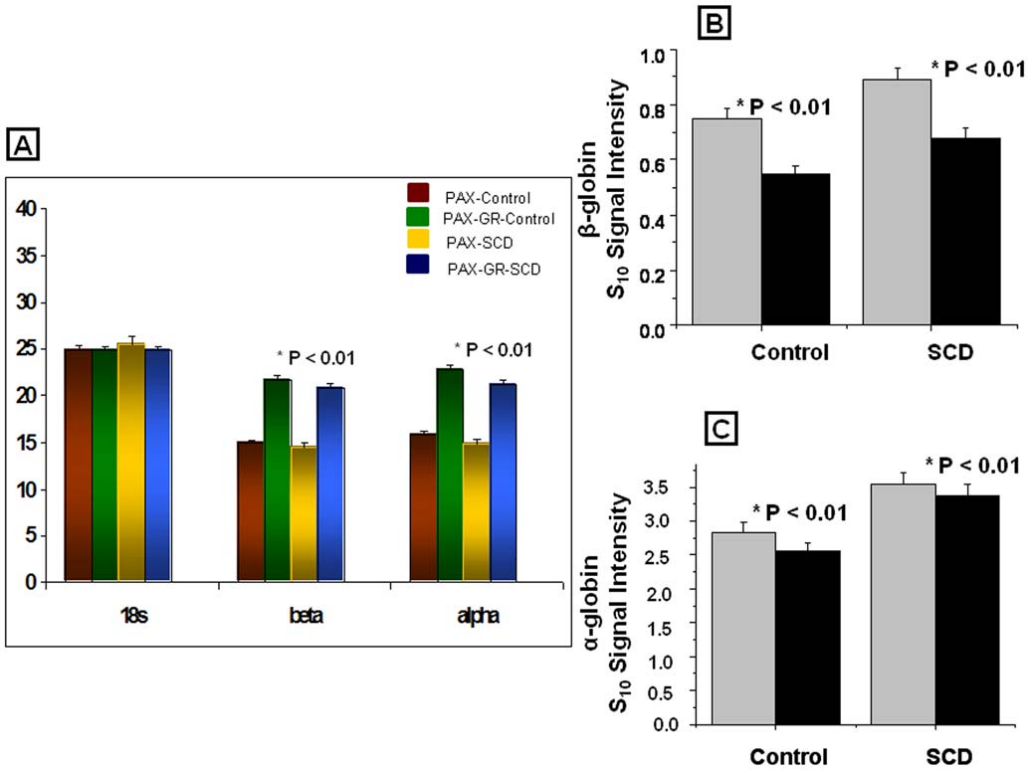
A. Expression of 18s, globin transcripts α, β by RT-PCR in PAX and PAX-GR from control and SCD samples. Ct values in the expression of globins are given as mean±SD for each group. Red bar - PAX-Control; Green bar - PAX-GR Control; Yellow bar - PAX-SCD; Blue bar - PAX-GR-SCD. B. Normalized signal intensities for β globin probe sets from microarrays for control and SCD samples. Values are given as mean±SD. Grey bar - PAX; Black bar - PAX-GR. C. Normalized signal intensities for α globin probesets from microarrays for control and SCD samples. Values are given as mean±SD. Grey bar - PAX; Black bar - PAX-GR.

To confirm the effectiveness of globin mRNA reduction, RNA samples from both PAX and PAX-GR samples were analyzed by RT-PCR using taqman primers for both alpha and beta globins and the normalized C_T_ values were calculated using 18s as the house keeping gene. There was significant depletion of both the alpha and beta globins in PAX-GR samples as represented by the increase in delta C_T_ values (normalized to 18S RNA) for both beta and alpha as a sequel to globin reduction as depicted in Figure 2A. Gamma globins did not show a significant difference between the PAX and PAX-GR samples. Examination of the S10 transformed signal intensity for the alpha and beta globins in the PAX and PAX-GR samples showed a similar pattern in the globin expression level with a signal intensity difference of >0.3 (or approximately log_10_ 2 fold representing about 3 fold on the S10 transform scale) between the PAX and PAX-GR for both the alpha and beta globin genes. (Figures 2B & C).

### Microarray analysis of expressed transcripts in PBMC, PAX and PAX-GR

**Figure 3:**
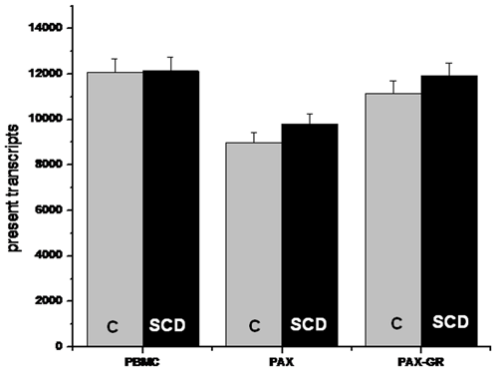
Detection of expressed transcripts in PBMC, PAX and PAX-GR in control (Grey bars) and SCD (Black bars) as determined by the present calls for the samples (n = 5) analyzed by microarrays. Values are given as mean±SD for the number of present transcripts in each of the groups in each sample type.

Upon hybridization of the biotinylated cRNA to HG U133 plus 2.0 gene chips, increased sensitivity in the detection of expressed transcripts was observed in PAX-GR samples as evaluated by the number of genes called above background (Present Calls), scaling factor, and signal intensity. Scale factor, an indirect measure of signal, with lower values indicating higher array signal decreased substantially from both control and SCD samples after globin depletion from 28±12.0 to 11.2±3.7. This thereby resulted in an increase in average signal and greater sensitivity throughout the distribution. Higher signal, especially for weakly expressed genes, resulted in better estimates of present calls. The highest number of transcripts was detected in the PBMC samples followed by PAX-GR and PAX respectively. Figure 3 shows this increase in detection sensitivity as evidenced by the increase in detectable transcripts after globin reduction in both control and SCD samples. Depletion of globins from both the control and SCD patient's samples increases the present call rate by 22–25% and this increase in detection sensitivity was more pronounced with the SCD samples.

In order to determine if globin reduction decreases intra sample variability a scatter plot analysis of all array elements was performed by comparing two control subjects on the 3 different sample types. PBMC samples showed the highest correlation (R^2^ = 0.92) and lowest variability between the samples while the PAX samples had an R^2^ of only 0.71. However, upon globin reduction this variability was reduced and the R^2^ value increased to 0.89. Further analysis of the genes expressed genes in either control and sickle cell disease samples using either the PAX and PAX – GR preparation showed that the median Mean Square Error (MSE) within group (SCD or control) to be 0.0063 for the PAX samples and 0.0037 for PAX-GR samples. This clearly demonstrates a substantial, greater than 40% reduction in the error variance, using the Globin reduction technique.

**Figure 4:**
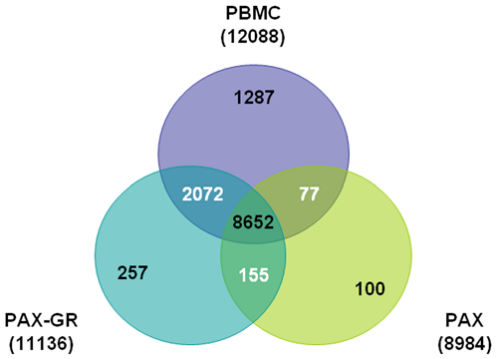
Venn diagram showing the relationship of present calls between PBMC, PAX and PAX-GR in SCD samples.

**Figure 5:**
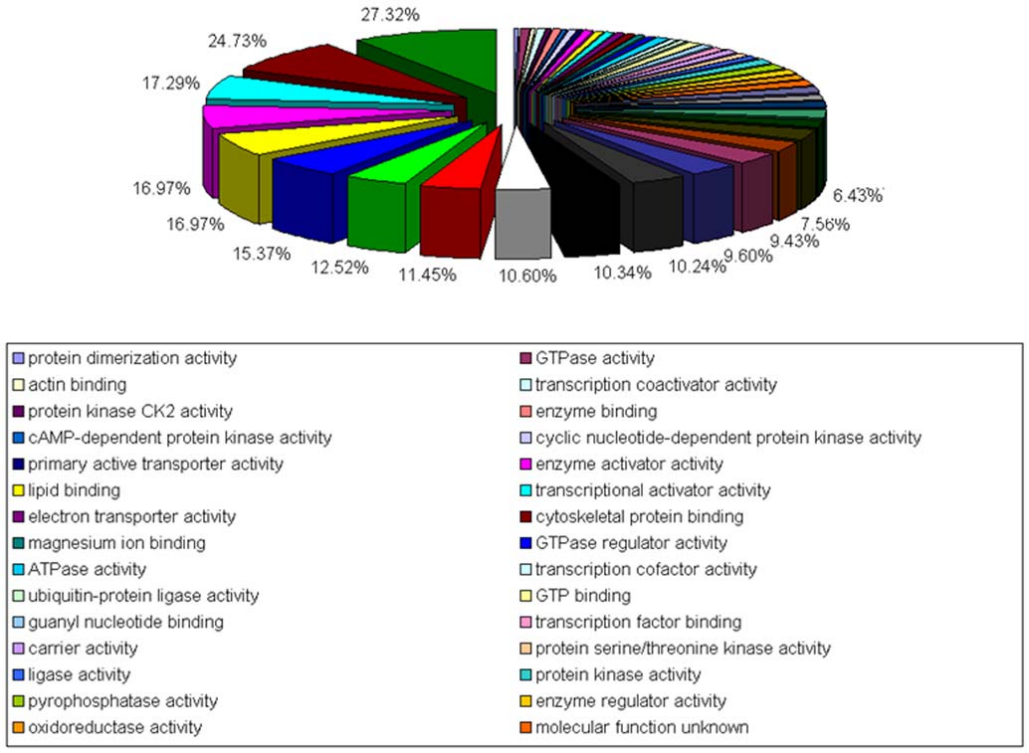
Gene ontology classification for genes common to PAX and PAX – GR. Many genes have multiple gene ontology descriptions.

A comparative analysis of expressed transcripts from the control and patient sample sets demonstrated a strong overlap of genes between the PAX-GR and PBMC with 11136 transcripts overlapping with 12088 transcripts from PBMCs. PAX samples on the other hand showed a lesser number (8954) overlapping with the PBMCs as shown in Figure 4. Similarly in SCD samples the overlap of transcripts between PBMC and PAX-GR was stronger with 10403 probe sets overlapping with 12134 transcripts in PBMCs than PAX with only 8208 probe sets overlapping with PBMCs. Between PAX and PAX-GR, the overlap was 88.8% in the control subjects and 94.4% in SCD patients suggesting that globin depletion process does not significantly alter the expression pattern of whole blood. Table 1 lists selected genes from this category of unaltered genes. GO analysis of genes that are common to both PAX and PAX-GR showed them to possess nucleic acid binding activity (27%), cytoskeletal protein binding (24.9%), transcription factor binding (17.9%) and enzyme activity (16.9%) as shown in Figure 5. Interestingly, globin depletion process unmasks 3,023 transcripts in the control samples and 5,644 transcripts from the SCD samples as evidenced by the ability to detect them as present calls in PAX-GR samples. Table 2 lists few selected genes that were unmasked by globin depletion process as evidenced by the transformation of an absent call to a present call along with few selected genes that remain unaltered. The unmasked genes examined by gene ontology analysis appear to function as binding proteins and enzymes involved in biological processes such as transcription, intracellular transport and biochemical signaling. The 100 genes representing hypothetical proteins, cDNA clones, unknown transcripts and genes such as hemochromatosis, hemoglobin epsilon 1 and transcribed locus similar to alpha hemoglobin that were detectable only in PAX samples were found to be masked or lost by globin reduction process as indicated by the inability to detect them in PAX-GR samples. BLAST analysis suggested that some of these transcripts may have sequence similarities to globins.

**Table 1:** List of selected genes that overlap between PAX and PAX-GR.

Cell Cycle Regulation/Apoptosis		
Probe set ID	Gene Title	Gene Symbol
201012_at	annexin A1	ANXA1
208796_s_at	cyclin G1	CCNG1
201095_at	death-associated protein	DAP
200046_at	defender against cell death 1	DAD1
225153_at	G elongation factor, mitochondrial 1	GFM1
201912_s_at	G1 to S phase transition 1	GSPT1
225153_at	G elongation factor, mitochondrial 1	GFM1
212508_at	modulator of apoptosis 1	MOAP1
210907_s_at	programmed cell death 10	PDCD10
**Cell Signaling**		
204312_x_at	cAMP responsive element binding protein 1	CREB1
219357_at	GTP binding protein 1	GTPBP1
223640_at	hematopoietic cell signal transducer	HCST
1552263_at	mitogen-activated protein kinase 1	MAPK1
1559052_s_at	p21 activated protein kinase 2	PAK2
219628_at	p53 target zinc finger protein	WIG1
203497_at	PPAR binding protein	PPARBP
225649_s_at	serine/threonine kinase 35	STK35
1552542_s_at	T-cell activation GTPase activating protein	TAGAP
**Oxidoreductase/Antioxidant/Stress response**		
203773_x_at	biliverdin reductase A	BLVRA
205950_s_at	carbonic anhydrase I	CA1
219933_at	glutaredoxin 2	GLRX2
200736_s_at	glutathione peroxidase 1	GPX1
218120_s_at	heme oxygenase (decycling) 2	HMOX2
200790_at	ornithine decarboxylase 1	ODC1
200844_s_at	peroxiredoxin 6	PRDX6
208864_s_at	thioredoxin	TXN
**Chemokines/Interleukins/Coagulation**		
200800_s_at	heat shock 70kDa protein 1A	HSPA1A
201656_at	integrin, alpha 6	ITGA6
204683_at	intercellular adhesion molecule 2	ICAM2
202531_at	interferon regulatory factor 1	IRF1
208200_at	interleukin 1, alpha	IL1A
204563_at	selectin L	SELL
203888_at	thrombomodulin	THBD
**Enzymatic Activity/Structural proteins**		
1554915_a_at	2′-phosphodiesterase	2′-PDE
200801_x_at	actin, beta	ACTB
204639_at	adenosine deaminase	ADA
201772_at	antizyme inhibitor 1	AZIN1
200068_s_at	calnexin	CANX
200946_x_at	glutamate dehydrogenase 1	GLUD1
200697_at	hexokinase	HK1
200650_s_at	lactate dehydrogenase A	LDHA
208980_s_at	ubiquitin C	UBC

**Table 2:** Representative Genes masked by globins and unmasked in PAX-GR.

Probe set ID	Gene Title	Symbol	Detection	
			PAX-GR	PAX
203271_s_at	unc-119 homolog (C. elegans)	UNC119	P	A
203431_s_at	Rho GTPase-activating protein	RICS	P	A
203702_s_at	tubulin tyrosine ligase-like family	TTLL4	P	A
203785_s_at	DEAD (Asp-Glu-Ala_Asp) box	DDX28	P	A
203834_s_at	trans-golgi network protein 2	TGOLN2	P	A
203890_s_at	death-associated protein kinase 3	DAPK3	P	P
204149_s_at	glutathione S-transferase M4	GSTM4	P	A
204278_s_at	estrogen receptor binding site associated	EBAG9	P	P
204292_x_at	serine/threonine kinase 11	STK11	P	A
204357_s_at	LIM domain kinase 1	LIMK1	P	A
208907_s_at	mitochondrial ribosomal protein S18B	MRPS18B	P	A
209202_s_at	exotoses (multiple)-like 3	EXTL3	M	A
210983_s_at	MCM7 minichromosome maintanence	MCM7	P	A
211370_s_at	mitogen-activated protein kinase kinase 5	MAP2K5	P	A
211513_s_at	opioid growth factor receptor	OGFR	P	A
213772_s_at	golgi associated, ARF binding protein 2	GGA2	P	A
214610_at	cytochrome P450, family 11, subfamily B	CYP11B1	P	M
216971_s_at	plectin 1	PLEC1	P	A
217770_at	phosphatidylinositol glycan, class T	PIGT	P	A
219646_at	Hypothetical protein FLJ20186	FLJ20186	P	A
219807_x_at	RAB4B, member RAS oncogene family	RAB4B	P	A

### Differential gene expression pattern in sickle cell disease

**Figure 6:**
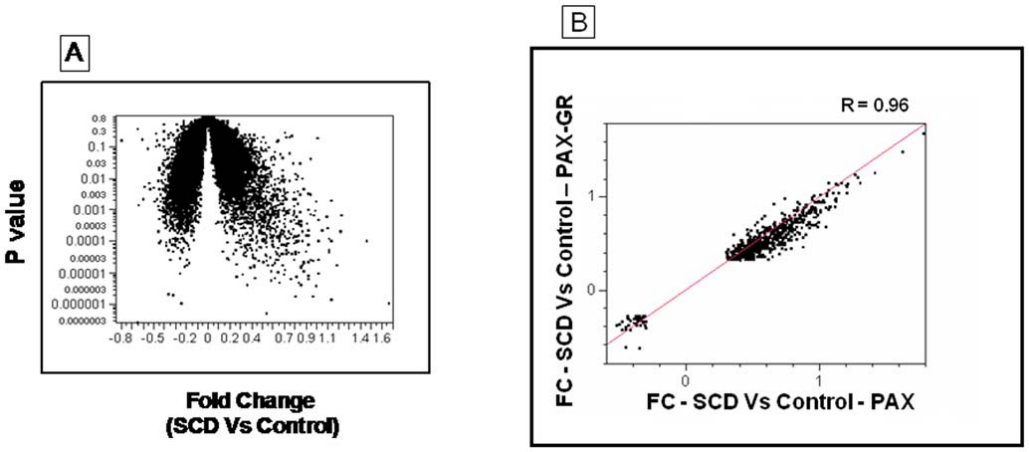
A – Volcano-plot of the comparison between SCD and control samples. The X axis represents the fold change and the Y axis represents the P value. B – Correlation of differentially expressed genes in PAX and PAX-GR samples.

While comparing the globin depleted whole blood gene expression profiles in patients with sickle cell disease and healthy African American volunteer subjects using stringent statistical filters of 20% false discovery rate and fold-change greater than 2, we identified 909 significantly altered transcripts (Figure 6A). These genes overlapped to a large extent with the differentially regulated gene list from the PAX group of samples, and the fold changes correlated strongly (R = 0.96) as shown in Figure 6B suggesting that the globin depletion process does not alter the original whole blood gene expression profile of these subjects.

**Figure 7:**
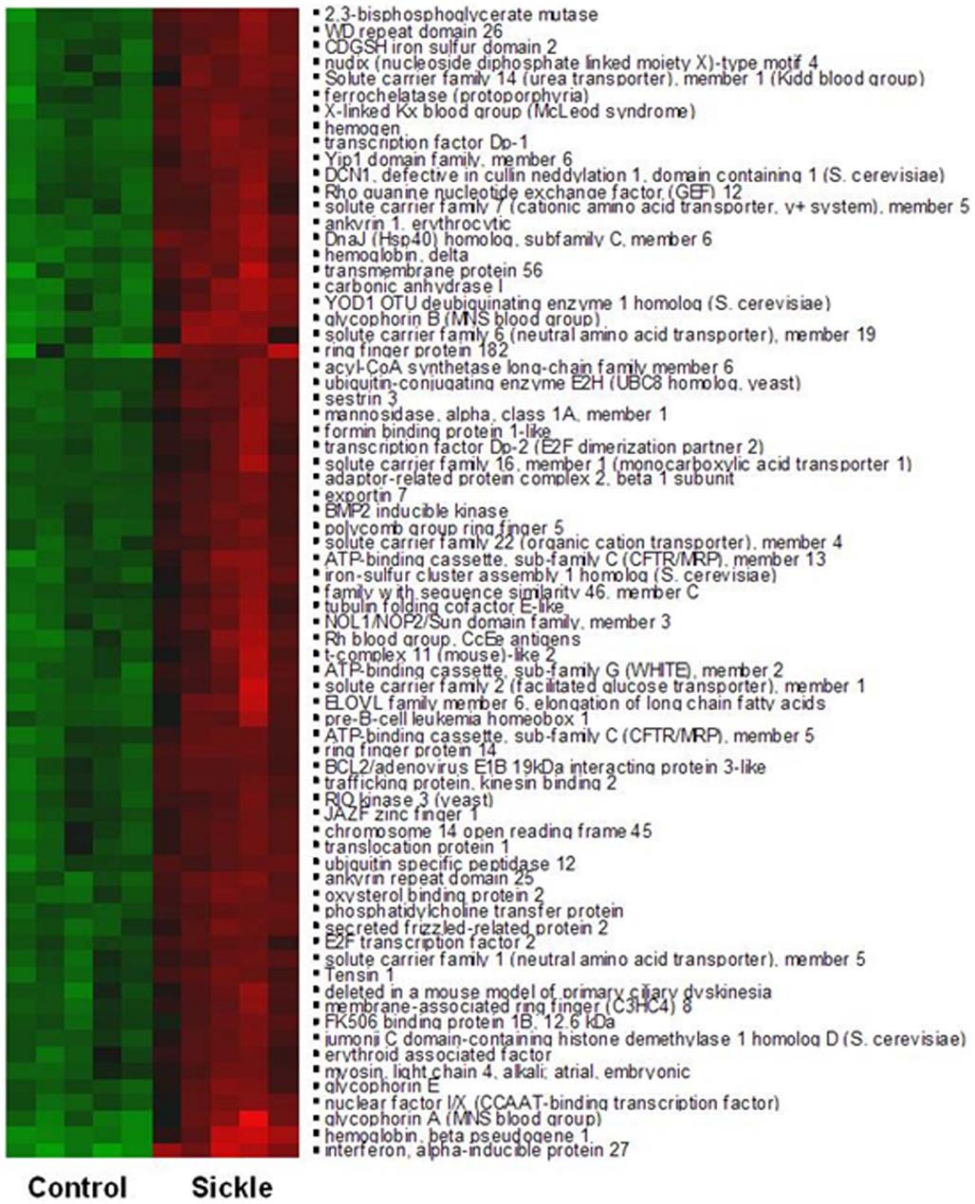
Heat Map of differential gene expression in PAX-GR in sickle cell disease (n = 5) in comparison to control subjects (n = 5). Cluster analysis was applied to gene expression data derived from all probes on HG-U133 plus 2.0 at FDR 10% and FC >5.0. The level of expression of each gene in each sample relative to the median level of expression of that gene across all samples is represented using a red, black and green color scale (green – below median; black – equal to median; red - above median). The dendrogram displays the unsupervised clustering of patients and control subjects using the differentially expressed gene list.

A closer examination of these differentially regulated genes after filtering at 10% FDR and FC greater than 5 as shown in Figure 7 revealed significant up regulation of red cell specific genes such as ankyrin, erythrocyte membrane protein band 3 and 4, hemoglobins and glycophorin A in sickle cell disease. Other transcripts that were identified to be differentially regulated in sickle cell disease included BMP2 kinase, selenium binding protein, exportin, spermine oxidase, peroxiredoxin 2, chemokines, interleukin receptor and peptidyl arginine deaminase and these are known to be key regulators in redox metabolism and inflammatory processes as shown in Table 3. Few selected genes from the list of differentially regulated genes with fold change >2, from the PAX-GR group were validated by RT-PCR analysis and the results that corroborate with the microarray data are shown in Table 4.

**Table 3:** List of differentially regulated genes in SCD from PAX-GR.

Probe set ID	Gene Title	Gene Symbol	FC
202411_at	interferon, alpha inducible protein 27	1F127	82.87
214407_x_at	glycophorin B	GYPB	22.75
216063_at	hemoglobin, beta	HBBP1	20.22
205950_s_at	carbonic anhydrase I	CA1	19.71
211821_x_at	glycophorin A	GYPA	17.65
203502_at	2,3-bisphosphoglycerate mutase	BPGM	16.58
203115_at	ferrochelatase (protoporphyria)	FECH	12.26
217799_x_at	ubiquitin-conjugating enzyme E2H	UBE2H	9.66
209301_at	carbonic anhydrase II	CA2	8.66
206834_at	hemoglobin, delta	HBD	7.90
59644_at	BMP2 inducible kinase	BMP2K	7.02
216984_x_at	Immunoglobulin lambda	IGLC2	6.48
225123_at	Sestrin 3	SESN3	6.15
212166_at	exportin 7	XPO7	6.09
201629_s_at	acid phosphatase 1	ACP1	5.50
202922_at	glutamate-cysteine ligase	GCLC	5.26
214433_s_at	selenium binding protein 1	SELENBP1	5.04
201060_x_at	stomatin	STOM	4.88
207793_s_at	erythrocyte membrane protein band 4.1	EPB4.1	4.70
202869_at	2′,5′-oligoadenylate synthetase 1	OAS1	4.63
212592_at	Immunoglobulin J	IGJ	3.43
211643_x_at	Immunoglobulin kappa	IGKC	3.22
210357_s_at	spermine oxidase	SMOX	3.19
205483_s_at	interferon, alpha-inducible protein	G1P2	3.16
39729_at	peroxiredoxin 2	PRDX2	3.07
240336_at	hemoglobin mu	HBM	3.00
223084_s_at	cyclin D-type binding-protein 1	CCNDBP1	2.86
204949_at	intercellular adhesion molecule 3	ICAM3	0.49
211982_x_at	exportin 6	XPO6	0.47
201041_s_at	dual specificity phosphatase 1	DUSP1	0.47
204174_at	arachidonate 5-lipoxygenase	ALOX5AP	0.42
226333_at	interleukin 6 receptor	IL6R	0.40
207094_at	interleukin 8 receptor, alpha	IL8RA	0.39
220001_at	peptidyl arginine deiminase, type IV	PADI4	0.33
210484_s_at	tumor necrosis factor receptor	TNFRSF10C	0.33
201087_at	paxillin	PXN	0.32
207008_at	interleukin 8 receptor, beta	IL8RB	0.32
204655_at	chemokine (C-C motif) ligand 5	CCL5	0.29
206515_at	cytochrome P450, family 4	CYP4F3	0.24

**Table 4:** Validation of microarray data by RT-PCR.

Gene Title	Symbol	Fold Change		P Value
		MA	RT-PCR	
Acid phosphatase 5	ACP5	4.09	5.66	<0.05
Arachidonate 5-lipoxygenase	ALOX5	−0.77	−0.82	<0.05
Carbonic anhydrase IV	CA4	−0.62	−0.56	<0.10
Ethanolamine kinase 1	ETNK1	2.58	3.12	<0.05
Glutamate-cysteine ligase	GCLC	5.02	6.24	<0.05
Hexokinase	HK1	3.52	3.96	<0.05

A comparative analysis of the differentially regulated genes from the 3 different sample types from this study to our previous study on PBMCs resulted in the mapping of 106 out of 112 probe sets from previously published data to the current data. Further analysis on the 106 probe sets showed a reasonably high correlation to the PBMCs from the current study with a correlation coefficient value of 0.51, followed by globin reduced PAX with a correlation coefficient value of 0.41. A poor correlation was observed with PAX samples with a correlation coefficient value of only 0.21. It has to be borne in mind that these studies were done in different batches using a totally different set of patients and control subjects and two different types of gene chips. Even considering these limitations, globin reduction improves the correlation to the PBMCs from our previous study suggesting the advantages of depleting globins in whole blood RNA for microarray studies.

## Discussion

Peripheral blood is an essential tissue type for biomedical research because of its critical roles in immune response and metabolism. The simplicity and ease of collection has also made peripheral blood an attractive surrogate tissue for the discovery of biomarkers of hematologic diseases and a wide range of nonhematologic disorders. Thus, applying microarray technology on peripheral blood may provide new insights of variations in global gene expression specifically associated with states of normal and disease and has the potential of applying the technology in disease detection and diagnosis.

Although global gene expression technology had been successfully applied on fractionated blood samples [10], [11] such as PBMCs, successful studies of gene expression profiles in whole-blood total RNA have been limited due to heterogeneous cell types and potential ex vivo changes from blood handling and processing. PBMCs with a more uniform cell population, containing lymphocytes and monocytes are the most transcriptionally active cells in blood [5] making it an ideal study specimen. However, the extra fractionation procedure for PBMCs requires a prolonged period before RNA stabilization, and this has been shown to have significant ex vivo changes in gene expression profiling [12], [13]. In addition, in multicenter clinical trials, isolation of PBMCs at the time of sample collection has been considered to be a major shortcoming as skilled technicians are needed for processing the samples at the site and this could also lead to operator induced variability in microarrys.

However, with the challenges unique to the whole blood sample, including complex composition of heterogeneous cell types and lower detection sensitivities and higher data variability, it is difficult now to apply microarray technology on whole-blood total RNA. It is widely believed that the abundant globin transcripts in whole blood are the causative factors for the lowered sensitivity and increased variability in microarray based gene expression studies. The current study is an exploratory research with the purpose of developing proper methods that would deplete hemoglobin RNA from whole blood, for applying microarray technology on globin depleted whole-blood total RNA. We chose a globin abundant system namely sickle cell disease for this study to evaluate the globin depletion process and also to discover novel genes that are associated with sickle cell disease including cell types such as nucleated red cells in addition to the conventional PBMCs.

Ambion's globin clear method resulted in 55–65 fold depletion of globin transcripts in whole blood and this ultimately led to a significant improvement in microarray data quality with 22–25% increase in the detection rate of expressed genes and this increase was more pronounced in SCD. This increase in detection sensitivity appears to be a reflection of higher signal intensity and lower signal to noise ratio as evidenced by the transformation of an absent call to a present call as shown in Table. 2. Interestingly, even after globin reduction the PAX and PAX-GR samples by principal component analysis clustered together rather than clustering with the PBMCs. More importantly, the basis of sample segregation appears to be dependent on sample types rather than the biological nature of the samples suggesting that PAX and PAX –GR even after globin depletion have a unique expression profile compared to PBMCs and this could be attributed to the presence of cells other than mononuclear cells in the whole blood RNA. The improvement in detection sensitivity after globin depletion also permitted detection of several genes that were masked by globins in the original PAX samples.

Previous studies on the effects of globin reduction from whole blood suggested that globin depletion can introduce transformations to original gene expression profile [13]. On the contrary, our comparative analysis on the different sample types as shown by the Venn diagram clearly demonstrated high fidelity in gene expression with a high degree of overlap (88–94%) between the PAX and PAX-GR samples. The globin depletion process appears to be advantageous as it reduces the variability of data between samples as shown by the scatter plot and the error analyses. More importantly, depletion of globins led to unmasking of more than 3000 transcripts in the whole blood samples which GO analysis indicates have biological process functions such as transcription, intracellular transport and biochemical signaling. This eventually led to an improvement in the degree of overlap between PAX-GR and PBMCs at 73–80%. The unmasking of genes by globin depletion process also led an increase in the identification of transcripts that are differentially regulated in sickle cell disease and these differentially regulated genes include red cell specific transcripts such as ankyrin, erythrocyte membrane protein band 3 and band 4, hemoglobins and glycophorin A. Transcripts such as BMP2 kinase, selenium binding protein, exportin, spermine oxidase, peroxiredoxin 2, chemokines, interleukin receptor and peptidyl arginine deaminase that are known to be key regulators in redox metabolism, inflammatory process also demonstrated differential regulation in sickle cell disease. This list of genes with statistical filters at 20%FDR and FC >2 differed considerably to the list obtained from PBMCs and this could be explained by the heterogeneous mixture of cells in whole blood. We then compared 112 differentially regulated genes (5% FDR and FC >1.2) from our previous study on PBMCs using a larger set of patients and controls to the current study by parametric analysis. By doing so, it was evident that PBMCs from the current study correlated to a large extent to the previous study with an R value of 0.51 followed by PAX-GR (R = 0.41) and PAX with an R value of only 0.21. Though these correlation coefficient values are not as high as one would expect them to be, it has to be remembered here that the two studies were performed using a completely different set of patients and volunteers using two different gene chips. Nevertheless, correlation studies clearly showed that globin reduction by unmasking several hundred genes in whole blood generates a gene expression profile that is moderately comparable to the expression profile of PBMCs.

This study thereby demonstrates that application of globin reduction process to whole blood is highly advantageous to generate meaningful data from microarrays. From a biological perspective, the globin depleted PAX gene versus the PBMC difference expression profile provided a window into real time erythrocyte expression profiles in vascular diseases In clinical investigations on hematological diseases such as sickle cell anemia, thalassemia, G6PD, pyruvate kinase deficiency and in malaria where the early red blood cell progenitors represent major contributors to pathophysiology, insights into the transcription profile of these cells may contribute greatly to our understanding of mechanism of disease, prognosis, and responses to therapeutics. The combination of globin mRNA reduction after whole-blood RNA stabilization represents a robust clinical research methodology for the discovery of biomarkers for hematologic diseases and in multicenter clinical trials investigating a wide range of nonhematologic disorders where fractionation of cell types is impracticable.
